# Numerical and Experimental Assessment of Oxygen Distribution in a Perfusion Bioreactor to Mimic the Osteochondral Niche

**DOI:** 10.1002/bit.70238

**Published:** 2026-05-20

**Authors:** Franziska Alt, Eric Langner, Hans‐Peter Wiesmann, Thomas Wallmersperger, Benjamin Kruppke

**Affiliations:** ^1^ Institute of Materials Science Technische Universität Dresden Dresden Saxony Germany; ^2^ Institute of Solid Mechanics Technische Universität Dresden Dresden Saxony Germany; ^3^ Institute for Organic and Macromolecular Chemistry Friedrich Schiller University Jena Jena Thuringia Germany; ^4^ Department of Biomaterials Institut für Bioprozess‐ und Analysenmesstechnik e.V., Rosenhof Heilbad Heiligenstadt Thuringia Germany

**Keywords:** bioreactor, experiments, finite element method, hypoxia, modelling, normoxia, numerical simulations, oxygen diffusion, oxygen perfusion

## Abstract

Oxygen supply is a critical parameter in 3D cell cultivation using bioreactors. Since bioreactor designs often prioritise practical constraints, understanding the oxygen supply dynamics of the media is crucial for achieving either uniform or spatially controlled oxygen delivery. This is particularly relevant for cultivating chondrocytes in hypoxic conditions. To evaluate oxygen supply, we used a concentric bioreactor with a central flow channel (140 mm length). Phosphate‐buffered saline (PBS) served as the medium, which was deoxygenated with nitrogen to create an oxygen sink. Oxygen diffusion in an 1.5% agarose gel bed was experimentally measured using fluorescence quenching oxygen detectors and compared to finite element simulation results. Additionally, two cavities in the gel were filled with collagen to mimic a cell‐free osteochondral niche. Measurements showed a gradual oxygen decrease with distance from the flow channel, reaching concentrations below 0.025 mM after 150 h. Reoxygenation occurred faster, with levels exceeding 0.15 mM within 50 and 80 h in setups without and with collagen, respectively. Simulation results initially matched experimental data only during initial or late stage of de‐/reoxygenation, but were improved by varying the diffusion coefficient and the mass transfer coefficient for liquid‐to‐gel transitions. Further refinements could include batch‐specific diffusion coefficients and concentration‐dependent diffusion adjustments. Understanding oxygen dynamics in bioreactors will enable precise oxygen delivery, particularly when integrating proliferating stem cells or chondrocytes as oxygen sinks. These findings pave the way for more effective bioreactor designs tailored to specific cell culture needs.

## 1 Introduction

The self‐repair capacity of articular cartilage is very limited in vivo, necessitating alternative approaches for effective clinical treatment. Tissue engineering (TE) has emerged as a promising method to generate transplantable cartilage in vitro suitable for repairing damaged areas. The functional development of tissue‐engineered cartilage is significantly influenced by the environment used during cultivation, wherefore bioreactors are the method of choice. Various bioreactors, including static or orbitally shaken petri‐dishes, concentric cylinder bioreactors, spinner flasks, rotating wall vessel (RWV), or perfusion bioreactors, have been employed in general biomaterials research and specifically cartilage TE studies (Kruppke~ 2020b; Smith et al. 2016; Fu et al. 2021; Vunjak‐Novakovic et al. 1999). Each type of bioreactor impacts the resulting cell development differently, with static cultures often yielding inhomogeneous matrix distributions and RWVs producing constructs with uniform cartilage formation and high extracellular matrix (ECM) content (Vunjak‐Novakovic et al. 1999). Generally, perfusion enhances matrix synthesis when exogenously added growth factors initiate chondrogenesis of mesenchymal stem cells and mechanical stimulation through fluid shear stress, hydrostatic pressure, and (intermittent) dynamic compression in bioreactors further promotes cartilage matrix biosynthesis (Dahlin et al. 2014; Fu et al. 2021; Lee et al. 2003; Carver and Heath 1999).

Increased strontium ion concentrations from degradable biomaterials also showed osteochondral tissue regeneration with a high proportion of chondrocytes in vivo (Kruppke et al. 2020a), with such studies also making it easier to estimate the ion concentration and residence times with the help of bioreactors.

The vast variability in bioreactor configurations causes numerical models to be crucial for comparing results across different studies and for optimising bioreactor designs and culture protocols (Cantarero Rivera and Chen 2022; Sacco et al. 2011; Sengers et al. 2008; Raimondi et al. 2011). Specific models are used to characterise the hydrodynamic environment around TE scaffolds, demonstrating that excessive fluid shear stress can lead to fibrous capsule formation, whereas low shear environments support chondrogenesis (Raimondi et al. 2002; Neitzel 2005). At the microscopic level, local fluid‐induced shear stress on chondrocytes has been evaluated. Besides mechanical factors, adequate nutrient supply is essential for the functional development of TE cartilage. Studies have modelled various aspects of transport and biological processes, including fresh medium supply in spinner flasks (Malda et al. 2004), fluid flow and oxygen transport in concentric cylinder bioreactors (Hossain et al. 2015; Sacco et al. 2011), as well as nutrient diffusion in biological systems (Travascio et al. 2020; Langner et al. 2023). Additionally, research work has examined the transport of ions in polymeric systems (Wallmersperger et al. 2007; Leo et al. 2005) and hydrogels (Leichsenring and Wallmersperger 2015) with coupled chemo‐electrical formulations, glucose and oxygen metabolism with lactate release (Travascio and Jackson 2017), the synthesis of extracellular matrix, and cell growth at the construct level (Jones and Chapman 2012). Despite these advances, cell behaviour remains a significant source of uncertainty in modelling studies, as often only very selective environmental conditions are considered for cell development. The evaluation of the complex cellular interplay in the osteochondral niche is usually not taken into account.

To gain a comprehensive understanding of the biochemical environment within TE constructs, it is necessary to model the key metabolite oxygen within a suitable bioreactor for both chondrogenesis and osteogenesis. Besides physical and biochemical cues, oxygen pressure plays a pivotal role in cell differentiation during skeletal development. This assertion is particularly evident in the context of endochondral ossification, a process that is intrinsically linked to the oxygen tension. Subsequent to a cascade of various processes, the mineralised bone is built starting from cartilage tissue and leaving the osteochondral tissue in the long bones. The fundamental process underlying this process of ossification and bone formation is also evident in the general differentiation behaviour of mesenchymal stem cells (Bahney et al. 2019; Mackie et al. 2008). Mesenchymal stem cells generally differentiate to bone cells in a relatively oxygenated environment, whereas chondrocytes reside in a hypoxic environment. This influence was shown for a bioreactor approach, where bone marrow‐derived stem cells were seeded in a collagen scaffold surrounded by an agarose bedding, to mimic oxygen diffusion through muscle tissue (Lee et al. 2017). The oxygen pressure was found to be a potent inducer of chondrogenesis in this system. Examination of the osteochondral niche showed that not only chondroitin sulphate A, the pH value, and systematically applied differentiation media regimes were potent variables for growth factor‐free differentiation approaches for chondrogenesis (Lee et al. 2019). The oxygen pressure (pO2) distribution in the bioreactor was also essential and showed to have a significant influence on cells in this artificial osteochondral niche. In this particular context, the bioreactor design that has been presented is principally intended for the purpose of simulating cartilage and endochondral tissue. However, it is also applicable to investigations on angiogenesis‐related tissue engineering approaches.

In vivo experiments frequently fail to address the question of the influence of the matrix materials on the oxygen supply to the cells, thus neglecting a key factor of chondrogenesis. Notwithstanding, the performance of the matrix materials, including collagen with hyaluronan or high‐sulfated chondroitin sulphate, as well as alginate‐based bio‐inks, has been demonstrated to be excellent (Foerster et al. 2017; Kilian et al. 2023). As demonstrated by Harris et al. monocyte chemotactic protein‐1 (MCP‐1), whose expression is subject to regulation by oxygen levels, exerts a suppressive effect on the process of chondrogenic differentiation of synovial mesenchymal precursor cells (Harris et al. 2013). As a consequence of this increased oxygen concentration, there was an increase in MCP‐1 expression in vitro, which resulted in a reduction in the differentiation of these cells into a cartilage‐specific lineage. The results of the study highlight the significance of oxygen's role in the regulation of cartilage formation via the MCP‐1 signalling pathway. In conjunction with the understanding of oxygen distribution and supply in hydrogel‐based tissue‐engineered cartilage constructs, these findings suggest novel methodologies for the control of regenerative processes. It is therefore imperative that precise knowledge of oxygen diffusion and distribution in commonly used hydrogels is obtained in order to ensure optimal cell nutrition.

This study aims to predict the distribution of oxygen within an adapted version of Lee's osteochondral‐niche bioreactor over time (Lee et al. 2017). The model will address several key questions: (i) whether oxygen supply is significantly affected by the fluid flow through the channel of the bioreactor, (ii) the mass transfer between the fluid and agarose, and (iii) the influence of material parameter variation on the overall system.

This paper is structured as follows: in Section 2, the design of the concentric cylindrical bioreactor, the process of the experiments (deoxygenation and reoxygenation), as well as the data measuring procedure are presented. These experiments are used to calibrate a model in Section 3. Numerical tests are conducted to gain understanding in the main transport processes within the system, such as the variation of the type of the fluid flow in the channel, the mass transfer coefficient and the diffusion coefficient. The comparisons between experiments and numerical simulations are described in Section 4. A conclusion of the work and an outlook for further investigations can be found in Section 5.

## 2 Experiments

### 2.1 Design of the Concentric Cylindrical Bioreactor

**Figure 1:**
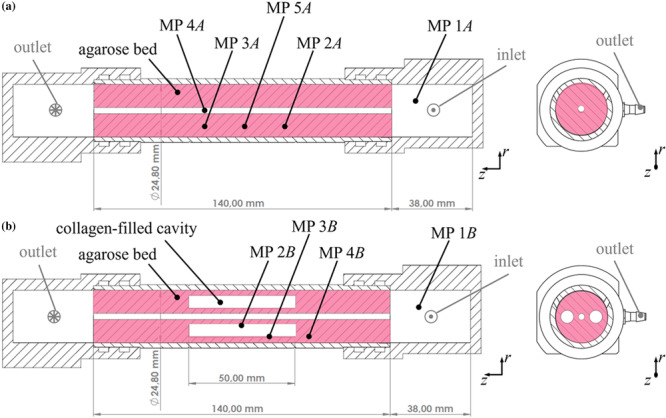
Schematic sectional views for the two different validation setups (denoted as Scheme a and b, respectively) with depicted oxygen sensor positions. The inlet is located on the right side with the measuring points MP 1A and MP 1B, respectively. The fluid flow goes from right to left.

A concentric cylindrical bioreactor was constructed using polycarbonate (PC), including a corpus of 140 mm length and 24.8 mm inner diameter. Agarose (ultra pure agarose; Invitrogen, USA) was dissolved in phosphate‐buffered saline (PBS; Sigma Aldrich, USA) (1.5 g/100 mL (m:v)) and melted above $90^{\circ }$C. The liquified agarose was poured into the corpus, creating a concentric perfusion channel of 3 mm diameter using a removable steel rod. After the agarose was solidified inside the corpus, two chamber attachments for the liquid perfusion inlet and outlet were attached to the corpus. These attachments were filled with PBS subsequently, and perfusion through the channel inside the agarose was performed. The inlet‐chamber is in the following referred to as ‘pre‐chamber’, indicating the direction of perfusion. The utilisation of silicon‐sealed holes facilitated the introduction of fibre‐based oxygen sensors (Oxford Optronix, UK), with the cylindrical corpus comprising six access points and the pre‐chamber one. The access points within the corpus were arranged in two longitudinal parallel lines of three holes each, distributed evenly over a length of 50 mm. The fibre‐based oxygen sensors were inserted in the system with Vasofix needle (20G, B.Braun, Germany). For validating the simulations, two setups were chosen. Scheme A includes five measuring positions (MP) inside the agarose bed as depicted in Figure 1a. For scheme B, two cavities inside the agarose bed were produced by temporary metal rods, as described for the liquid channels before, and filled with col I (rat tail; Corning, USA) during bioreactor preparation (Figure 1b).

The perfusion rate of $5.5\;{\;\text{mL min}\;}^{-1}$ was ensured by means of a peristaltic pump (ISMATEC, Postnova Analytics GmbH, Germany), which was positioned after a PBS reservoir and in advance to the pre‐chamber. After being pumped through the concentric cylinder bioreactor, the PBS was redirected to the reservoir.

### 2.2 Oxygen Control in the System

Experiments were conducted to validate the performed numerical simulations. For investigating oxygen diffusion, an oxygen potential had to be created, wherefore the oxygen in the system was removed first. This process is referred to as ‘deoxygenation’. The concentric cylindrical bioreactor was prepared in terms of filling with 1.5% agarose, inserting the fibre‐based oxygen sensors, and placing it in the HypoxyLab chamber (Oxford Optronix, UK), ensuring a defined atmosphere around the bioreactor, tubings, and liquid reservoir. Atmospheric settings were chosen to be 5 mm Hg oxygen, 0% ${\;\text{CO}\;}_{2}$, 42% relative humidity, and $22^{\circ }\;C\;$. Meanwhile, the PBS reservoir was rinsed with gaseous nitrogen for at least 20 min. Oxygen partial pressure was measured to be 0 mm Hg afterwards. The deoxygenated PBS was placed in the HypoxyLab as well, and the bioreactor was filled at maximum pump speed. After ensuring that the bioreactor was completely filled with deoxygenated PBS, the defined perfusion rate of $5.5\;{\;\text{mm min}\;}^{-1}$ was set. The deoxygenation of agarose inside the bioreactor was monitored using fibre‐based oxygen sensors, inserted at respective locations. During deoxygenation, the PBS reservoir was removed from the HypoxyLab and rinsed with gaseous nitrogen every 2–3 days to ensure the lowest possible oxygen content. After approx. 1 week, the oxygen partial pressure of the agarose was found to be 8 mm Hg, which was considered to be the minimal possible partial oxygen pressure.

The oxygen transport from the oxygen‐rich PBS towards the agarose is referred to as ‘reoxygenation’, for which deoxygenated PBS was completely removed from the centric cylindrical bioreactor and the Hypoxylab atmospheric setting of oxygen was changed to 150 mm Hg. The medium reservoir was exchanged to oxygen‐containing PBS and the bioreactor was completely filled with it at maximum pump speed, while oxygen partial pressure was recorded by the inserted fibre‐based oxygen sensors in parallel.

### 2.3 Data Acquisition and Calculation of Oxygen Concentration

For the purpose of measuring oxygen levels, the OxylitePro XL (Oxford Optronix, UK) was employed, comprising four channels. To facilitate the acquisition of data, an in‐house Arduino‐based data acquisition system (DAQ) was utilised. The DAQ converted the specified voltage from the OxylitePro into the corresponding partial oxygen pressure in mmHg. The data were recorded at 20‐min intervals at the outset of the experiment, with the interval subsequently adjusted to 1, 2, or 4 h in accordance with the progressive duration of the experiment. Between different time points, the OxylitePro was powered down.

**Figure 2:**
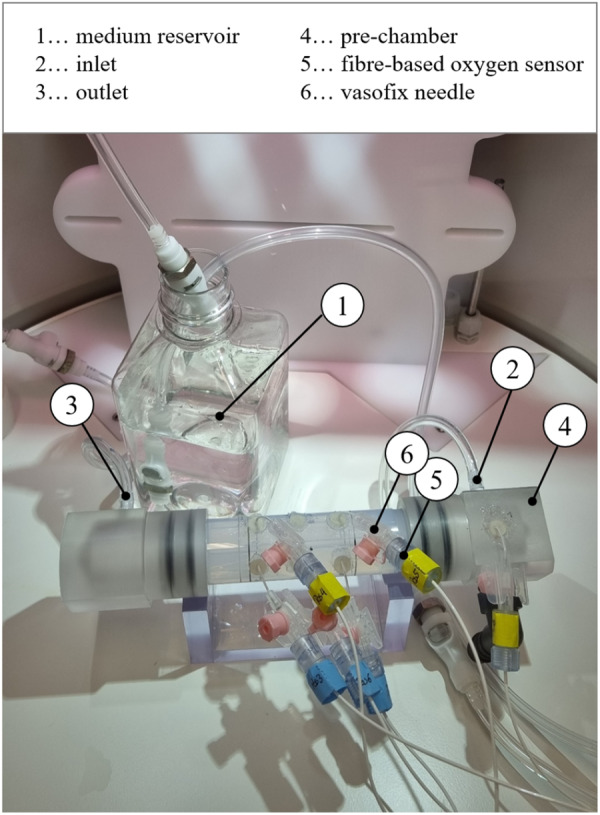
Photography of the cylindrical bioreactor inside the Hypoxylab with inserted oxygen sensors according to scheme A, see Figure 1a.

The OxylitePro XL device offers four channels for data acquisition. With five distinct positions within the concentric cylindrical bioreactor employed for oxygen measurement, each sensor was attached to its designated measuring position (Figure 2). The sensor location within the corresponding reading channel on the OxylitePro XL was subsequently adjusted, yielding a constant acquisition of oxygen values for four MPs, while MP 1A was intermittently tracked.

In order to allow comparisons with the simulation, it was necessary to consider the oxygen concentration ($c_{O_{2}}$) in mM rather than the partial oxygen pressure ($p_{O_{2}}$) in mmHg. The solubility of gases in liquids depends on the specific type and composition of the liquid in question and is described by the Bunsen constant. Given that the salt concentration in PBS is similar to that of cell culture medium, the Bunsen constant of $1.26\;\mu {\;\text{M O}\;}_{2}$ per 1 mmHg was taken at first approximation. This is described in the following equation:

(1) $$c_{O_{2}}\approx p_{O_{2}}\cdot 1.26\times 10^{-3}\frac{\;\text{m M}}{\text{mmHg}\;}.$$

### 2.4 Statistics

At each time point, the DAQ acquired 20 measurements distributed over a 5‐min period. The mean of the 20 measurements was calculated to represent a single time point, with the standard deviation serving as an error bar. The reproducibility of the measurement procedure was ensured by the repeated measurement of each design configuration on two occasions. However, it should be noted that only a single measurement of each design configuration is presented.

## 3 Numerical Simulations

### 3.1 Modelling of Oxygen Transport

In order to describe the oxygen distribution within the bioreactor over time, the continuity equation is considered:

(2) $$\dot{c}+\nabla \cdot J-r\left(c\right)=0$$

where c is the concentration of oxygen molecules, J the flux term, r the reaction term, and ∇ the nabla operator and $\dot{\left(\;\;\right)}$ the time derivative of a quantity. In this work, r is assumed to be zero as no chondrocytes or other cells are present in the system and thus, no oxygen can be consumed or produced. The transport of oxygen within the bioreactor is described by various transport phenomena that need to be taken into account for the flux J: (i) diffusion – the transport due to a concentration gradient, (ii) convection – the transport due to a velocity field within the channel, and (iii) a mass transfer between the channel that is filled with a liquid, containing dissolved salts (phosphate buffered saline (PBS)) and the agarose (see Section 2). Therefore, the flow of oxygen can be expressed mathematically via the constitutive law:

(3) J=−D(x)∇c+vc

where D(x) is the position‐dependent diffusion coefficient and v the velocity of the fluid. The partial differential equation (PDE) is solved with the finite element method (FEM). Here, the weak formulation is needed as a starting point:

(4) $$\int_{\Omega }\dot{c}\;\delta c\;\;d\;V+\int_{\Omega }D\left(x\right)\nabla c\cdot \nabla \delta c\;\;d\;V-\int_{\Omega }c\nabla \delta c\cdot v\;\;d\;V$$

(5) $$-\int_{\partial \Omega }D\left(x\right)\nabla c\cdot n\delta c\;\;d\;A+\int_{\partial \Omega }cv\cdot n\;\delta c\;\;d\;A=0.$$

In addition, the implicit Euler‐backward time discretisation scheme $\dot{c}=\frac{1}{\Delta t}\left(c^{n+1}-c^{n}\right)$ is used with Δt being the time step size and n being the current time step. The symmetry of the domain was exploited, and a quarter of the total domain was spatially discretised with tetrahedral finite elements (see Figure 7). Near the collagen‐filled cavity (see Figure 1b), the FE mesh is finer to allow the resolution of high concentration gradients between two materials near the interface. As boundary conditions (BCs), either (i) Dirichlet boundary conditions (DBCs) $c\left(x\right)=c_{D}\;\forall x\in \partial \Omega_{D}$ with prescribed concentrations of oxygen $c_{D}$ at the boundary $\partial \Omega_{D}$, (ii) Neumann boundary conditions (NBCs) $-D\nabla c\cdot n={\bar{j}}_{N}\;\forall x\in \partial \Omega_{N}$ with a prescribed flux ${\bar{j}}_{N}$ normal to the boundary $\partial \Omega_{N}$, or (iii) Robin boundary conditions (RBCs) $-D\nabla c\cdot n=\beta \left(c-c_{\infty }\right)\;\forall x\in \partial \Omega_{R}$ as a mixture of the first two BCs with β being the mass transfer coefficient and $c_{\infty }$ the concentration near the boundary $\partial \Omega_{R}$ can be set. In Section 3.2, it is evaluated which BCs lead to the best agreement between experimental and numerical investigation. Furthermore, the time‐dependent PDE is solved with the open‐source finite element tool FEniCSx (Baratta et al. 2023).

### 3.2 Numerical Testing

In this section, several numerical modifications of the system are described in order to agree well with the experimental procedure in Section 2. It contains (i) two different flow types that prescribe the velocity v within the channel and (ii) the difference between DBCs and RBCs at both the interface between the channel and agarose and the surface facing the pre‐chamber of the bioreactor (see Figure 1).

#### 3.2.1 Effect of Flow

The flow of fluid or perfusion rate of $5.5\;{\;\text{mL min}\;}^{-1}$ is driven by a peristaltic pump in z‐direction. Since we do not solve Navier–Stokes equations within the channel and thus the velocity field, two different analytical expressions for v are evaluated and compared.

Assuming a constant velocity over the radial coordinate r of the channel and measuring the volumetric flow rate $\dot{V}$, an averaged velocity v in pipe direction can be calculated as

(6) $$v_{z}=\frac{\dot{V}}{\pi R^{2}}.$$

A volumetric flow rate of $\dot{V}=5.5{\;\text{mL min}\;}^{-1}$ was set, that is, a velocity of $v\approx 12.97\;{\;\text{mm s}\;}^{-1}$ was obtained for a radius R=1.5mm. That means that the radius‐independent velocity leads to a uniform distribution of the concentration measured at MP 1A/4A or MP 1B. Since the liquid also flows relatively fast through the channel in relation to the channel length (140mm) and the timescale is several hours, the concentration c of oxygen along the channel can be assumed to be constant. Therefore, the perfusion rate is considered to have no influence in the given order of magnitude.

Another analytical and more realistic approach is the assumption of the Hagen–Poiseuille flow within the channel (Sutera and Skalak 1993). Here, the fluid flows laminar in a parabolic shape where the velocity v is zero at the interface between the channel and the agarose. This means that the concentration is not directly transported to the interface, but needs to diffuse within the channel in radial direction. The pressure gradient in z‐direction can be expressed by

(7) $$p_{,z}=-\frac{8\dot{V}\eta }{\pi R^{4}}$$

with η being the dynamic viscosity. The velocity in pipe direction can be expressed by (Baehr and Stephan 2010)

(8) $$v_{z}\left(r\right)=-\frac{1}{4\eta }p_{,z}R^{2}\left[1-{\left(\frac{r}{R}\right)}^{2}\right].$$

Inserting Equation (7) in Equation (8) leads to

(9) $$v_{z}\left(r\right)=\frac{2\dot{V}}{\pi R^{2}}\left[1-{\left(\frac{r}{R}\right)}^{2}\right].$$

Both velocities (Equations (6) and (9)) were incorporated separately into the model for the transport due to convection, see Equation (3). The results were compared to zero flow behaviour, that is, the concentration in the channel does not depend on the coordinate. Due to the long experimental timescale, numerical test results do not show changes in the transport behaviour of oxygen for these two different flow assumptions compared to no flow. This leads to the conclusion that convection in the fluid channel does not have to be taken into account for such a perfusion rate, but that the concentration within the channel can be assumed to be the concentration measured at MP 4A or MP 1B.

#### 3.2.2 Mass Transfer

As already discussed in Section 2, the concentration of oxygen within the channel is measured at MP 4A and MP 1B. In these numerical tests, it is assumed that the same constant and time‐dependent concentration is present in the pre‐chamber and in the channel of the bioreactor. The use of time‐dependent DBCs both at the interface between the channel and the agarose and at the surface facing the pre‐chamber leads to a faster transport of oxygen into the medium than observed in the experiments. Therefore, it is necessary to include RBCs with a mass transfer coefficient β in analogy to a heat transfer coefficient (Baehr and Stephan 2010). In general, β depends on the type of flow (laminar/turbulent), on the properties of the fluid and solid, on the geometrical form of the system and on the concentration difference itself. However, β is unknown and needs to be adjusted, such that the numerical results are in agreement to the experimental results. Therefore, different values for β were used and the Euclidean error ϵ between experiments and simulation for MP 5A was evaluated with

(10) $$\epsilon =||c_{\text{exp,MP}\;5A}-c_{\text{sim,MP}\;5A}|{|}_{2}.$$

The minimum Euclidean error ϵ was used to determine $\beta =1\times^{-3}{\;\text{mm s}\;}^{-1}$. The environmental concentration $c_{\infty }$ that is used for RBCs needs to be adjusted according to the concentration measured at MP 4A and MP 1B, respectively.

## 4 Results

In this section, different experimental procedures are conducted and compared with numerical results: (i) the deoxygenation process of pure agarose in Section 4.1, (ii) the reoxygenation process of pure agarose in Section 4.2, and (iii) the reoxygenation process of collagen embedded in agarose in Section 4.3. In these three sections, a diffusion coefficient of $D=0.2589\times 10^{-2}\;{\;\text{mm}}^{2}{s\;}^{-1}$ was used for 1.5% agarose by extrapolating the measured diffusion coefficients by Hulst et al. of $D=0.256\times 10^{-2}{\text{mm}}^{2}s^{-1},D=0.199\times 10^{-2}{\text{mm}}^{2}s^{-1}$, and $D=0.168\times 10^{-2}{\;\text{mm}}^{2}{s\;}^{-1}$ for 2%, 5%, and 8% agarose, respectively (Hulst et al. 1989). Furthermore, a mass transfer coefficient of $\beta =1\times^{-3}{\;\text{mm s}\;}^{-1}$ was employed. An effect on the results due to variations of D and β is discussed in Section 4.4.

### 4.1 Deoxygenation of Pure Agarose

**Figure 3:**
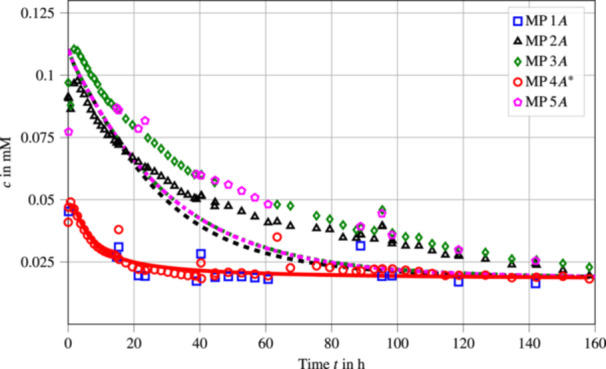
Experimental and numerical results for deoxygenation process in pure agarose: plot of the oxygen concentration c vs. time t for experimental values (symbols) and numerical values (solid and dashed lines) at the different measuring points according to Figure 1a. The experimental results of MP 4A have been used for calibrating the environmental concentration $c_{\infty }$ (marked by 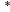).

The deoxygenation shows a steady progression of the decreasing oxygen content in the chamber over 158 h (Figure 3). Two different deoxygenation processes can be recognised. In MP 1A and MP 4A, there is only an oxygen concentration of 0.05 mM at the beginning, which is reduced to the achievable minimum of 0.02 mM at 40 h. This rapid drop is due to the fact that MP 1A and MP 4A are located in the pre‐chamber and in the liquid channel in the bioreactor. This ensures that the medium previously containing oxygen is quickly flushed out by the nitrogen‐purged and therefore oxygen‐free medium. After reducing the oxygen concentration in the pre‐chamber to its minimum level, c remains almost constant. As oxygen is released from the agarose to the fluid in the channel, a slightly higher oxygen concentration is present at the outlet. However, this is not measured directly, but anticipated from MP 4A (see Figure 1). The interrupted measurement point sequence from MP 1A is a result of the fact that the OxylitePro's number of channels is limited to four. Therefore, the measurements at MP 1A were recorded by manually starting the instrument and reconnecting it during the measurement pauses of the automated readouts. The other three measuring points MP 2A, MP 3A, and MP 5A, which are located in the agarose, show a curve that starts from a significantly higher level from hour 1 (0.1 and 0.11 mM, respectively) and then decreases continuously until values below 0.025 mM are reached after 150 h, which corresponds approximately to the values in the pre‐chamber and in the channel. The concentration in MP 2A is always approx. 0.01 mM below the values in MP 3A and MP 5A. The latter two, however, lie almost on an identical curve. This can be explained by the fact that MP 2A is significantly closer (ca. 45 mm) to the inlet of the liquid channel and the pre‐chamber, while MP 3A is significantly further away (ca. 90 mm) from the start of the channel and the pre‐chamber. A comparison of the numerical results shows that the transport of oxygen is slightly faster. In addition, the MPs inside the bioreactor are almost identical as the main transport originates from the channel, especially at the beginning of the simulation time. Furthermore, the same initial values at t=0 for these MPs were prescribed for this simulation.

### 4.2 Reoxygenation of Pure Agarose

**Figure 4:**
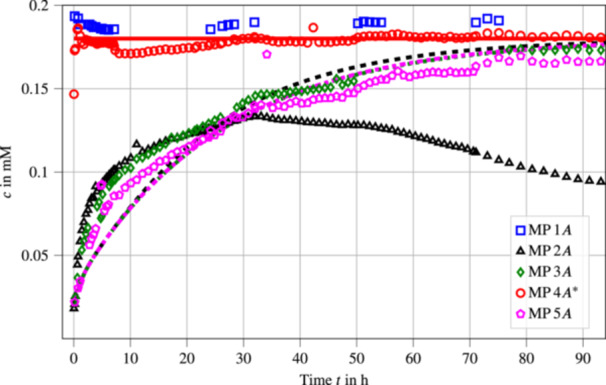
Experimental and numerical results for oxygenation process in pure agarose: plot of the oxygen concentration c vs. time t for experimental values (symbols) and numerical values (solid and dashed lines) at the different measuring points according to Figure 1a. The experimental results of MP 4A have been used for calibrating the environmental concentration $c_{\infty }$ (marked by 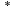).

Starting from the almost completely deoxygenated bioreactor, the (re‐)oxygenation can be traced particularly well in the in vitro experiment, as well as with in silico simulations. Once again, it can be seen that the two positions MP 1A and MP 4A in the PBS solution adjust rapidly to the initial level for the atmospheric oxygen partial pressure, that is, approx. 0.19 mM (Figure 4). It is noticeable that MP 1A shows the highest oxygen concentrations over the entire course, while the concentration in MP 4A, which is located in the channel, is approx. 0.02 mM lower.

As expected, the oxygenation of the agarose filling in the bioreactor is significantly delayed. MP 2A shows a faster increase in oxygen content up to 24 h compared to MP 3A and MP 4A, which is due to the closer position of MP 2A to the inlet and the pre‐chamber. In the beginning, the simulation reveals that the oxygen transport into the medium is rather slower than the real measured values. After approx. 10 h, the oxygen transport is slightly too fast compared to the experiments. In addition, the numerically determined values at the MPs 2A, 3A, and 5A are very similar. A small difference to MP 2A arises after some time, since the transport process from the channel is superimposed on that from the pre‐chamber.

After 24 h, the oxygen concentrations of the three MPs A in the agarose coincide. It is noticeable that MP 3A and MP 5A continue to show a continuous, but significantly slower increase in oxygen concentration and have almost reached the level of the PBS channel (MP 4A) after about 90 h. The slow reoxygenation of the agarose is most likely explained by the limited contact area between the flow channel and the gel, which restricts the overall oxygen flux and results in a prolonged equilibration time of oxygen within agarose. The results for the simulation are close to the experimental measurements for MP 1A and MP 5A for this time range. However, MP 2A shows an almost linear decrease in oxygen concentration from 32 h onwards. Here it can be assumed that there is a local contamination of the bioreactor, resulting in an oxygen sink. The growth of the bacteria under oxygen consumption not only appears to prevent the actually expected increase in oxygen concentration (according to the simulation, as well as the trend for MP 3A and MP 5A), but the oxygen concentration that has already built up around MP 2A is continuously reduced. It is to be expected that the concentration will reach a constant level that corresponds to a balance between the oxygen supplied from the channel and the consumption by the bacteria. Excessive consumption would lead to a reduction in the bacteria and in turn to the establishment of an equilibrium. The oxygen decrease observed here is (unintentionally) of great significance, as it demonstrates that the oxygen sink at MP 2A does not affect the increasing oxygen content of MP 5A compared to MP 3A, which suggests a higher oxygen transport in radial direction rather than in longitudinal direction. In agarose, the formation of bacterial biofilms occurs in discrete pockets, as opposed to the formation of dense biofilms (Pabst et al. 2016). Pabst et al. observed a decline in the bacterial growth rate to less than 10% of its maximum value, thus demonstrating the bacterial dependency on oxygen supply. Since our system provides oxygen in the whole agarose bed, bacteria should have enough oxygen supply to migrate to different areas. Instead, our findings reveal a local effect of bacterial oxygen consumption at MP 2A as no discernible influence could be measured at adjacent MP 5A. Combining this observation with the findings of Pabst et al. we are assuming that the agarose is limiting the diffusion/migration capacities of the bacteria. This is even further consolidated by a comparison between MP 5A and adjacent MP 3A, revealing no significance in the measured oxygen values. Based on that, it can be continually concluded that oxygen supply is guaranteed in the radial direction rather than the longitudinal direction.

The local dependence of oxygen availability and the replenishment of the oxygen consumed can be demonstrated particularly clearly with these investigations. Nevertheless, it is important to emphasise that the oxygen consumption of bacteria cannot be directly translated or compared with that of cells, such as chondrocytes or osteoblasts. The observation permits solely the interpretation of the direction of oxygen supply.

### 4.3 Reoxygenation of Collagen Within Agarose

**Figure 5:**
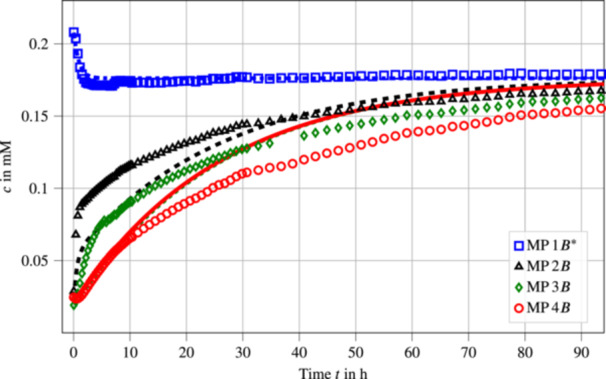
Experimental and numerical results for reoxygenation process with collagen: plot of the oxygen concentration c vs. time t for experimental values (symbols) and numerical values (solid and dashed lines) at the different measuring points according to Figure 1b. The experimental results of MP 1B have been used for calibrating the environmental concentration $c_{\infty }$ (marked by 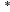).

At first glance, it is apparent that the oxygenation of the bioreactor with collagen‐filled cavities (Figure 5) and the bioreactor filled with agarose solely (Figure 4) is very similar. Nevertheless, the collagen filling is particularly interesting in detail, as it shall mimic the osteochondral niche in future. As a rough approximation, the channels in the bioreactor are modelled as blood vessels, the agarose as muscle or connective tissue and the collagen as the extracellular matrix of the bone and cartilage cells, in order to be able to investigate the influence of the oxygen partial pressure on cell differentiation in the niche in future. To do this, the state of the oxygen supply without cells must be analysed in detail so that optimal, as well as deficient conditions can be set precisely for the cell culture at a later stage. Based on the curve shape, a very rapid increase in oxygen can be seen within the first hour at MP 2B, the MP that lies directly on the channel‐side boundary of the collagen reservoir. In the same period, a comparatively strong drop in oxygen concentration can also be seen in the pre‐chamber (at MP 1B), which, according to the MPs A, can be equated with the channel. After that, a constant level is reached at MP 1B and the oxygen content of MPs B also rises continuously. The initial drop of oxygen at MP 1B represents the interaction between the oxygenated PBS and the oxygen‐free system. It is evident that, due to the sensor's strategic positioning within the medium, this particular measuring point is capable of rapidly capturing even minor alterations. The oxygen‐rich medium interacts with the oxygen‐depleted system, thereby releasing oxygen. This effect is particularly evident within the initial 2 h. The medium is circulated through a closed circuit, thereby ensuring that it is only able to absorb oxygen from the reservoir and through the silicone tubes. The stable level of MP 1B after 2 h indicates that a balance has been reached between the supply of oxygen from the medium to the agarose bed and the enrichment of the medium with oxygen. The oxygen concentration in the collagen at MP 2B is consistently higher than at MP 3B, which is further away from the channel. The favoured oxygen diffusion in collagen is shown by the sustained oxygen concentration at MP 4B, which has the same distance from the channel as MP 3B but is completely surrounded by agarose. After 90 h, only MP 2B is almost at the level of the pre‐chamber, although all MPs exceeded the concentration of 0.15 mM after 40 h (MP 2B), 60 h (MP 3B), and 80 h (MP 4B), respectively. An essential parameter for describing mass transfer between two immiscible phases at equilibrium is the partitioning coefficient, which expresses the ratio of the solute concentrations (in this case, oxygen) in both phases (in this case, agarose and collagen). Both materials are hydrogels with a high water content. In view of the fact that oxygen is a small, non‐polar molecule, it is anticipated that its solubility primarily depends on the water content. Based on this, we assume that the partitioning coefficient of oxygen between agarose and collagen is close to unity and can therefore be neglected in the present context. However, macromolecules such as growth factors or serum proteins can adsorb to collagen or be partially excluded from the agarose network, thereby changing their local availability and transport dynamics. In the context of future experiments and simulations, the incorporation of material‐specific partitioning coefficients for such molecules holds considerable potential in facilitating more realistic predictions of nutrient and signalling gradients, particularly within the domain of 3D cell culture systems. However, it should be noted that this aspect falls outside the scope of the present study.

The simulated oxygen concentration curves generally only show suitable predictions in partial areas. For MP 2B, for example, the rapid increase in the first hour is clearly evident, but due to the assumed diffusion and mass transfer coefficients, the dashed black line at the beginning is more at the level of MP 3B, which is far away from the channel in the collagen. The curves only begin to match towards the end of the measurement and the numerical simulation, but they indicate an oxygen level even closer to the pre‐chamber concentration. The simulation for MP 3B and MP 4B follow almost identical paths, which can be attributed to the fact that for collagen and agarose in the literature with about $D=1.7\times 10^{-5}\;{\;\text{cm}}^{2}{s\;}^{-1}$ to $D=2.5\times 10^{-5}\;{\;\text{cm}}^{2}{s\;}^{-1}$ for collagen at 3 and 1 weight percent, respectively (Colom et al. 2014), and $D=1.7\times 10^{-5}\;{\;\text{cm}}^{2}{s\;}^{-1}$ to $D=2.6\times 10^{-5}\;{\;\text{cm}}^{2}{s\;}^{-1}$ for agarose of 8 to 2 weight percent almost identical oxygen diffusion coefficients are given for the selected polymer concentrations (Hulst et al. 1989). Since no transition coefficients at the hydrogel interfaces were assumed in the model either, the predicted oxygen levels during the oxygenation and finally after 90 h are identical.

For the observed discrepancy between experimental and simulated oxygen diffusion rates during reoxygenation in the agarose, as well as in the collagen gel, various factors could influence the actual diffusion coefficient (D) of oxygen, which might explain why the measured oxygen increase is faster than expected. The literature provides insights into parameters that could affect D, particularly temperature, solvent composition, and gel concentration.

Temperature has a well‐documented impact on diffusion rates, generally increasing D with rising temperature due to increased molecular kinetic energy. In a bioreactor setting, even small fluctuations in temperature can significantly accelerate oxygen diffusion. This might not always be accounted for the simulations that assume a stable temperature. For instance, Elsayed et al. reported that the diffusion coefficient of oxygen in water is $D=2\times 10^{-5}\;{\;\text{cm}}^{2}{s\;}^{-1}$ (Elsayed et al. 2014), but this could vary slightly with temperature changes typical in dynamic culture environments, especially under constant liquid flow conditions and measurements over several nights and days, potentially leading to more rapid oxygen uptake in real‐time measurements.

The composition of the solvent from which oxygen diffuses into the agarose in which gels are immersed, such as phosphate‐buffered saline (PBS) in this case, also plays a critical role. In isotonic saline, for example, Place et al. measured a diffusion coefficient of $D=2.84\times 10^{-5}\;{\;\text{cm}}^{2}{s\;}^{-1}$ at $37^{\circ }$C, but when serum proteins were added, D decreased to approximately $D=2.69\times 10^{-5}\;{\;\text{cm}}^{2}{s\;}^{-1}$ (Place et al. 2017). In aqueous alkaline and acidic solutions, the oxygen diffusion coefficients were investigated by Baur and also showed a wide scatter between 1 M KOH solution ($D=1.6\times 10^{-5}\;{\;\text{cm}}^{2}{s\;}^{-1}$) and $0.1\;{\;\text{M Na}}_{2}{\text{SO}\;}_{4}$ solution ($D=2.5\times 10^{-5}\;{\;\text{cm}}^{2}{s\;}^{-1}$) (Baur 2007). Protein and ion concentrations may thus alter the diffusivity, potentially lowering it in certain simulations, if PBS alone is used as a reference. In practice, ions and macromolecules could either impede or enhance D depending on their specific concentrations and interactions with oxygen molecules.

Furthermore, the concentration of gel components such as collagen and agarose in PBS also has a substantial influence on oxygen diffusivity. Higher gel concentrations typically reduce D due to a denser matrix that restricts oxygen movement. Cheema et al. found that compressed cylindrical collagen scaffolds at 11% density exhibited a diffusion coefficient of $4.5\times 10^{6}\;{\;\text{cm}}^{2}{s\;}^{-1}$, while higher densities (e.g., 34%) lowered D to $1.7\times 10^{6}\;{\;\text{cm}}^{2}{s\;}^{-1}$ (Cheema et al. 2012). Similarly, Hulst et al. reported decreasing D values with higher agarose concentrations of which the extrapolated $D=0.2589\times 10^{-2}\;{\;\text{mm}}^{2}{s\;}^{-1}$ ($D=2.589\times 10^{-5}\;{\;\text{cm}}^{2}{s\;}^{-1}$, (Hulst et al. 1989)) for 1.5% agarose was used for the experimental setup. They report oxygen diffusion coefficients of agarose and agar hydrogels. The relative diffusion coefficients with respect to those in water decreased to 92.4% for agarose and 59.2% for agar at $30^{\circ }$C, respectively.

In numerical simulations, if idealised or averaged diffusion coefficients are used, they may not reflect the real‐time dynamics of variable gel densities, potentially explaining the faster reoxygenation observed in experimental setups where local concentration variations impact oxygen mobility more directly. Additionally, only a linear dependence of the oxygen transport J on the concentration gradient ∇c was used, as no literature data was available. However, it is possible that nonlinear constitutive models need to be parametrised to have more accurate predictions of the oxygen distribution over time.

Considering these factors, the faster‐than‐expected increase in measured oxygen levels may be attributed to the combined effects of temperature fluctuations, specific solvent interactions with dissolved oxygen, and heterogeneous gel concentrations, which all contribute to higher real‐time diffusion rates than those suggested by static simulations. In solids, systems have been described whose diffusion coefficient depends on the concentration of the diffusing substance, which is negligible only below 0.1% (Mylona et al. 2014). In liquids and gels, this dependence of the diffusion coefficient has not been described so far and has been limited to the influences of temperature, Boltzmann constant, dynamic viscosity of the solvent and hydrodynamic radius of the diffusing particles according to the Stokes‐Einstein equation (Einstein 1905). In our view, two main parameters emerged with regard to this interim conclusion, which were varied in the following section for better adaptation of in silico and in vitro results. Future simulations might consider incorporating these dynamic variables to more accurately predict oxygen reoxygenation behaviour in bioreactor‐cultured gels.

### 4.4 Effect of Parameter Variation

**Figure 6:**
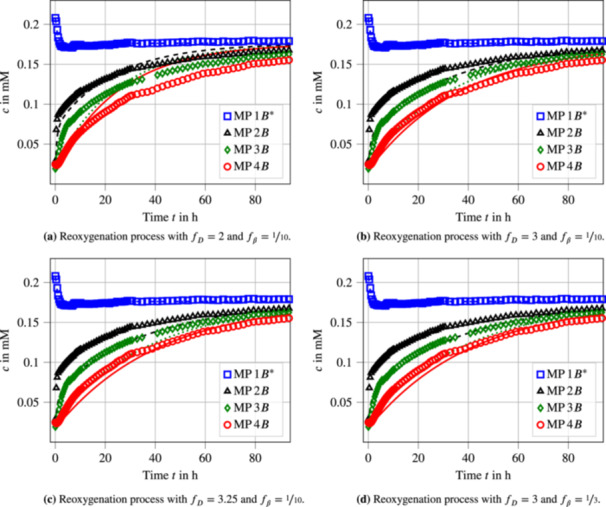
Evaluation of the effect of parameter variation during the reoxygenation process with collagen within the cavity embedded in agarose: plot of the oxygen concentration c vs. time t for experimental values (symbols) and numerical values (solid and dashed lines) at the different measuring points according to Figure 1b. The experimental results of MP 1B have been used for calibrating the environmental concentration $c_{\infty }$ (marked by 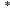).

In the following, the effect of varying parameters in the model on the results is analysed. The diffusion coefficient and the mass transfer coefficient were varied by $D^{*}=D∕f_{D}$ and $\beta^{*}=\beta ∕f_{\beta }$. As the results for the reoxygenation process with collagen in Figure 5 show, (i) the diffusion process in the simulation is too fast, as the concentration in the immediate environment MP 1B is reached too early, and (ii) the mass transfer coefficient is too low, so that the increase at the beginning of the experiment cannot be achieved. Therefore, the original diffusion coefficient is decreased and the original mass transfer coefficient increased. Results for exemplary parameter variations are shown in Figure 6.

**Figure 7:**
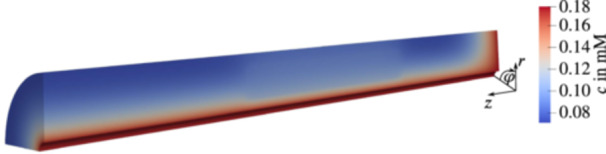
Exemplary oxygen distribution according to the numerical simulation in Figure 6c at t=20h.

Comparing these different parameter variations, a factor for the diffusion coefficient $f_{D}=3.25$ and for the mass transfer coefficient $f_{\beta }=1∕10$ leads to a very good agreement, especially in MP 4B. However, it is noticeable that the drastic increase at the beginning of the experimental process can be better approximated by higher diffusion coefficients with, for example, $f_{D}=2$ which in turn would be too high at larger times t. This indicates that the oxygen transport may not only depend linearly on the concentration gradient, but rather nonlinear. In addition, the variation of β also affects the result, see Figure 6b,d, in particular at the beginning of the experiment. However, this influence is not as strong as the variation of D. The oxygen distribution c(x) for a certain time t=20h is plotted in Figure 7. The transport of the oxygen into agarose takes place at both the interface to the channel and the interface to the pre‐chamber at z=0. However, the diffusion length towards the cavity from the channel is smaller than from the pre‐chamber. A closer look into the cavity filled with collagen reveals that the oxygen concentration within this medium is higher, as the diffusion coefficient for collagen was not changed for these investigations.

**Figure 8:**
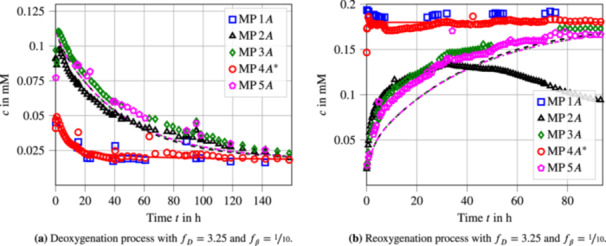
Evaluation of the effect of parameter variation during the reoxygenation and deoxygenation processes with pure agarose: plot of the oxygen concentration c vs. time t for experimental values (symbols) and numerical values (solid and dashed lines) at the different measuring points according to Figure 1a. The experimental results of MP 4A have been used for calibrating the environmental concentration $c_{\infty }$ (marked by 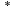).

If these parameter variations are transferred to the experiments carried out with pure agarose, the concentration curves shown in Figure 8 are obtained. For the deoxygenation process (Figure 8a), the simulations are in an excellent agreement with the experiments, especially at smaller times. At larger times, the diffusion process slows down. The same behaviour can be seen for the reoxygenation process in Figure 8b. However, the diffusion process at the beginning of this experiment is too low to match the experimental measurements well. These two observations lead to the same hypothesis as before: The diffusional transport and also the mass transfer coefficient might be affected non‐linearly by the concentration gradient. Considering the large number of publications on the diffusion process of oxygen in hydrogels and also its observation in bioreactors with a continuous oxygen supply via a perfusing medium, it is unfortunately rarely or never considered to what extent not only the diffusion itself, but also the mass transfer of oxygen from the liquid into the hydrogel is limiting the oxygen supply (Figueiredo and Pace 2018; Sachlos and Czernuszka 2003; Chin et al. 2008; Pozuelo et al. 2014; Elsayed et al. 2014; Lee et al. 2017). This seems particularly critical because the cultivated cells are usually referred to directly act as a barrier to oxygen diffusion. Thus, in addition to the diffusion quotient, they are considered to be the boundary of the supply depth (Figueiredo and Pace 2018), which in turn depends on the gel concentration. Thus, only an interplay between cell number and gel concentration is discussed here, which in turn requires a trade‐off between desired gel strength and good oxygen diffusion. Based on our findings, a transition coefficient between cell culture medium and hydrogel (and possibly also between different hydrogels) should be considered in the future.

## 5 Conclusion and Outlook

In this work, we focused on the oxygen transport and distribution over time within a bioreactor. Since a better understanding of the distribution of the molecules is required for further studies on cell growth, various experimental studies were carried out in pure agarose and with collagen embedded in the osteochondral niche. A numerical model was established to elucidate the effects of the transport mechanisms (such as the fluid flow and diffusion) and influence of material parameters.

The flow‐through bioreactor setup used in the experiment showed relatively long deoxygenation and reoxygenation times. It was clear that the liquid supply (whether with nitrogen‐saturated PBS or oxygen‐containing PBS) and the channel in the centre of the bioreactor maintained a very constant oxygen‐poor or oxygen‐rich level for the entire duration of the experiment. For the deoxygenation, a complete reduction of the oxygen content in the agarose cannot be assumed under 120 h. In contrast, reoxygenation already reaches a high level after approx. 80–90 h. This is particularly relevant for future experiments with oxygen sinks, that is, cells, in order to set cell number‐specific supply situations, considering that the cell numbers usually present in cartilage are lower than those used in vitro in cell culture and tissue engineering. Thus, the aim is not to consider cell‐related oxygen reduction, but to use the information gained here to specifically adjust reduced oxygen pressure to provide optimal conditions for chondrocytes.

Considering all experimental and numerical investigations, it is notable that the model allows to mimic the osteochondral niche as it is possible to predict the oxygen distribution within the whole bioreactor with a very good agreement to the experimental data. However, it is not sufficient to vary the coefficients β and D to have a perfect alignment between experimental and numerical values over the whole time. This leads to the conclusion that, if it is absolutely essential to have a better prediction, then, it is necessary to extend the numerical model. For instance, the linear constitutive law, that is used in this study, can be replaced by more complicated material behaviour. This would exemplary allow a diffusion process that depends on both the magnitude of the concentration and the non‐linearity of the concentration gradient.

A comparison of the literature showed large differences in the diffusion coefficients depending on the type and concentration of the hydrogel. Consequently, we also conclude that the batches, manufacturers, or polymer provenances have a relevant influence on hydrogels from natural resources. In particular, when researching more complex hydrogel mixtures, or biphasic materials, or as shown in our setup, the replication of cartilage tissue (collagen) in a surrounding capsule (alginate) as a diffusion barrier, a thorough analysis of long‐term oxygen supply is indispensable. As a consequence, the oxygen supply of chondrocytes in larger hydrogel scaffolds should be investigated, whereby the in vitro and in vivo investigations must be supported by meticulous simulations. Other factors, such as the complex geometry, for example, of the intervertebral discs or the menisci, or the mechanical compression of the gels and the resulting changes in diffusion and, possibly, material transport processes, must be taken into account. Only then, the development of large‐volume cartilage replacement materials will be successful in the future.

## Author Contributions

**Franziska Alt:** writing – original draft, writing – review and editing, visualisation, data curation, conceptualisation, methodology, investigation. **Eric Langner:** writing – original draft, writing – review and editing, visualisation, data curation, formal analysis, software, conceptualisation, methodology, formal analysis, investigation. **Hans‐Peter Wiesmann:** writing – review and editing, conceptualisation, funding acquisition, supervision, resources. **Thomas Wallmersperger:** writing – review and editing, conceptualisation, funding acquisition, supervision, resources. **Benjamin Kruppke:** writing – original draft, writing – review and editing, supervision, conceptualisation, methodology, funding acquisition.

## Conflicts of Interest

The authors declare no conflicts of interest.

## Data Availability

The data that support the findings of this study are available from the corresponding author upon reasonable request.
